# Upon the Entire Resection of Both Superior Maxillary Bones

**Published:** 1853-09

**Authors:** 

**Affiliations:** Professor of Clinical Surgery at Erlangen


*Upon the Entire Resection of both Superior Maxillary Bones.* By M. Heyfelder, Professor of Clinical Surgery at Erlangen.—Gensoul at Lyons, and Lizars in England, were the first to extirpate (1827) the superior maxillary bone of one side in cases of disease. Since then the operation has been often repeated, and in 1846, Ried (*Die Resectionem der Knochen*) was able to collect thirty-five cases, of which twenty-four were followed by success. If to these be added the cases of Morel-Lavalle, Maisonneuve, Chaissaignac, Robert, and Muston, together with those related in the Surgical Cliniques of Munich, Wurzburg, and Erlangen, not yet published, above fifty cases may be easily enumerated. The same remark cannot be made of the resection of both superior maxillæ. M. Velpeau accords the honor to Rogers, "who, in 1824, took away the upper jawbone of both sides as far as the pterygoid processes, and who found it scarcely necessary to divide the lip." This description is too brief to be intelligible. Liston removed the entire superior maxillæ of one side, and part of the other. Dupuytren (1818) effected the partial removal of both maxillæ in a man sixty-eight years of age.

Thus, the cases hitherto known are but examples of the partial resection of both maxillary bones.

The complete extirpation of both bones has been performed by Heyfelder twice. The first operation was in the summer of 1844; the second, in January, 1850.

*Case* 1.—A. Schmidt, aged 25, came to the Clinique, June 13th, 1844, suffering from a tumor of the face, which, from his account, had commenced a-year ago, in the posterior part of the palate, and had gradually involved both superior maxillary bones. The nose was pushed upwards, and flattened; the palatine arch was depressed towards the tongue; the face was affected with œdematous swelling; both respiration and deglutition were impaired, speech was embarrassed, and the sleep broken. The teeth, though loosened, were sound; only two incisors were wanting. The tumor appeared everywhere hard, uneven, and insensible to the touch, and did not pass beyond the boundaries of the superior maxillary bones. The constitution was good; lancinating pains had been felt in the tumor only during the last few weeks.

Dr. Heyfelder concluded that the tumor was of an indolent malignant character, and that the only remedy consisted in the entire removal of both maxillary bones. The operation was performed, June 23, 1844. The patient being seated in a chair, the head resting against the chest of an assistant, two incisions were made from the external angles of the eyes in the labial commissures, and the included parts were reflected upwards to the internal angles of the eyes, and to the ossa nasi. The flap, thus formed, was raised towards the forehead, until the infra-orbital ridge was exposed. Then the chain-saw of Jeffray was passed through the spheno-maxillary fissures, and the molar bones were divided; the maxillæ were next separated from the ossa nasi; the vomer and the thinner bones were cut with strong scissors; after which a chisel, applied with moderate force to the superior part of the tumor, was sufficient to effect its separation. The accessions of syncope prolonged the operation, which, however, did not last longer than three quarters of an hour. Very little blood was lost; torsion and compression sufficed to arrest the hæmorrhage. Two hours afterwards, the edges of the wounds, from the angles of the eyes to the corners of the mouth, were united by twenty-six points of suture, and cold lotions were applied; there was no reaction nor swelling; the patient could swallow water and broth. Four days after the operation it was remarked, that the wounds had become almost entirely united by first intention. In six weeks the patient was presented at the Physico-Medical Society of Erlangen, and on August 25 he was discharged.

The following was his condition :—There was no deformity of the features : in his mouth there was seen along the median line a fissure thirteen lines long and three lines broad ; the extirpated parts had been replaced by the tissue of a cicatrix, firm and solid at the circumference, but somewhat softer near the fissure; the soft palate and the uvula were in their natural place; deglutition was free, and the tongue in a better state than formerly; the nose had resumed its usual form and direction ; the face, which, before the operation, was monkey-like, once again possessed a human expression.

The microscopical examination of the tumor showed that it was of cancerous nature. Six months afterwards, the patient, in good health, went to work in the fields; but in the summer of 1845, Dr. Heyfelder was informed that another tumor was forming in the forehead.

*Case* 1.—Martin Lochner, aged 55, was affected in the upper lip, twelve years ago, with a cancerous growth, which was operated upon three years after its appearance. For two years there was no return ; but, subsequently, a small tumor developed itself near the cicatrix, approached the nose, and excited violent pains. Soon a cancerous ulcer formed, which extended over the right half of the upper lip, the ala nasi, and the palate. The patient came into the hospital Jan. 21, 1850, in the following state :—A horrible cancerous ulcer, commencing at the right commissure of the mouth, occupies the greater part of the upper lip, and has destroyed both the right ala and the septum nasi. All the parts were covered with painful, bleeding, fungous growths. The palatine arch was converted into an irregular, knobbed, cancerous mass; most of the teeth were lost; an offensive secretion was constantly flowing, and the patient was thinned and worn out.

The operation was performed January 24. Two incisions were made, exactly as in the preceding case. The chain-saw was used to separate the left os malæ, and Liston's bone forceps to divide the right. The remains of the septum nasi and the vomer were cut with strong scissors. A considerable quantity of the soft parts was likewise taken away. The patient was fed by a syringe. The left coronary artery bled three hours after the operation, but it was easily tied. The case went on well; and, on February 18, when the patient was presented at the Physico-Medical Society, the following was his condition:—The greater part of the wounds were united; but a deficiency existed in the face, corresponding to the disease, at the superior part of which were seen the vertical portion of the ethmoid, and the two ethmo-turbinal bones. In the velum palati there was an opening, two centimetres wide. The destruction of the lip and of the nose produced on the right side a triangular opening, the base of which corresponded to the mouth, and the superior angle to the root of the nose.

He can take liquid food, but his speech is unintelligible; but, when the opening in the palate is closed by the sponge, he can make himself understood. Autoplastic operations were unadvisable, from the exhausted condition of the man.—*Med. Times & Gaz. from Rev. Medico-Chirurgicale de Paris.*

